# Functional Bracing in a Femur Non-union Following Fracture Related Infection: A Case Report

**DOI:** 10.5704/MOJ.2411.011

**Published:** 2024-11

**Authors:** K Nazirul-Mubin, MY Nazri, S Ahmad-Fadzli

**Affiliations:** Department of Orthopaedics, Traumatology and Rehabilitation, International Islamic University Malaysia, Kuantan, Malaysia

**Keywords:** non-union, functional brace, fracture related infection, limb reconstruction, bone healing

## Abstract

Non-union refers to a disruption in the process of fracture repair, which can be identified through sequential clinical and radiographic assessments. The distinction between septic and aseptic non-union is essential because the treatment strategies are fundamentally different. Non-unions are most often treated surgically as it helps to provide both mechanical stability and good biological environment to promote bone healing. However, there is also the option of managing it conservatively by proper immobilisation using functional brace which is an alternative for surgical procedures and widely reported in tibia non-union cases. To date, there has been no reported case of femur non-union successfully treated with a functional brace. This case report details the success of treatment using a functional brace in a mentally disabled gentleman who sustained a femur non-union following a fracture related infection.

## Introduction

The American Food and Drug Administration (FDA) has established a widely accepted standard definition of non-union, which refers to a fracture that persists for at least nine months, without evidence of healing for a consecutive period of three months. The choice between operative and non-operative treatment depends on various factors including the type, the location and stability of the non-union, the patient's overall health, and their individual circumstances. Non-operative treatment is generally considered for non-unions with a good chance of healing without surgery. There are multiple cases reported involving delayed union and non-union of the tibia that successfully obtained fracture union after six months application of functional brace^1^. But there were little to none reported successful cases of treating femur non-union with a functional brace.

We report an unusual case of a femur non-union secondary to fracture related infection, which was successfully treated with a femoral functional bracing.

## Case Report

A 46-year-old male diagnosed with Down syndrome presented to our hospital with a chief complaint of left thigh pain. He sustained an intertrochanteric fracture of the left femur from a motor vehicle accident six years ago, for which he underwent an open reduction and internal fixation (ORIF) with proximal femur locking plate. He was asymptomatic for six years.

**Fig. 1:**
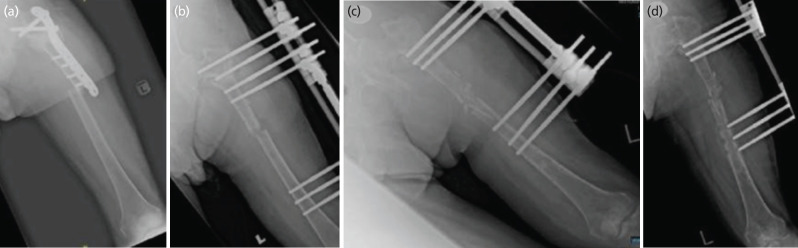
AP radiograph of Left femur showing; (a) peri-implant fracture following fall, (b) post fracture stabilisation with monolateral fixation system, (c) post fracture augmentation with non-vascularised fibula graft, (d) loosening of LRS distal pins.

A few hours prior to consult, the patient fell and experienced pain over the left thigh. Radiographs revealed a peri-implant fracture over proximal left femur (Fig. 1a). Subsequently he was planned for removal of implant, and stabilisation of the fracture site with an interlocking nail. Unfortunately, intra-operatively we noted purulent material around the implant. We decided to proceed with radical debridement of the fracture site and surrounding soft tissue, removal of implant and high tibial pin insertion. Interestingly, the fracture edges were found to be still healthy by evidence of good bleeding and was not sclerotic. Staphylococcus aureus organism was isolated from the two deep tissues from the mentioned surgery which was sensitive to Oxacillin and Eryhthromycin confirming a diagnosis of fracture related infection (FRI) over the left femur. We started the patient on IV Cloxacillin as targeted intravenous antibiotics and the fracture was stabilised with a monolateral fixator system which was done about three weeks following the trivial fall (Fig. 1b). The monolateral external fixator (LRS) was planned as a definitive management for the FRI as it was considered stable enough to allow fracture healing. However, at three months follow-up, no signs of fracture healing was seen on radiographs (i.e: callus formation). Hence, we counselled the patient and family for intervention by bone grafting, but they were not keen and would rather continue observing until nine months.

At nine months follow-up, radiographs did not show signs suggestive of bone healing. Fortunately, his septic markers were all normal. Therefore, we diagnosed him as atrophic femur non-union, and he underwent an ipsilateral non-vascularised fibula bone grafting over the non-union site with readjustment of the monolateral fixation (Fig. 1c). We opted for a non-vascularised fibula bone graft as we felt the surrounding soft tissue envelope was healthy enough to provide adequate blood supply to the fracture site and this type of graft provides good osteoconductive and additional stability to the fracture site. Opting for a vascularised fibular graft would only cause unnecessary complications to the patient and would require a plastic surgeon's expertise which was not available in our hospital. Post-operatively he was allowed for partial weight bearing ambulation. Later, however his condition was complicated with pin-tract infection and loosening of distal pin sites during his third month follow-up (Fig. 1d). Clinically, haemoserous discharge was detected from the distal pin sites with loosening (Checkettes-Ottenburg Grade 4). We proceeded with pin site debridement and removal of all pins; however, a microbiological sample was not obtained during surgery as oftentimes pinsite infections will grow multiple organisms and the result may not be reliable. At our centre, the infection status of debrided pin site infections was determined by the clinical appearance of the wound and patient symptoms. Therefore, following the debridement, we started him on broad-spectrum IV antibiotics for one week followed by oral antibiotics for five weeks. Unfortunately, intra-operatively we noticed that the fracture site was still mobile but still decided to remove the pins due to the risk of worsening infection.

**Fig. 2:**
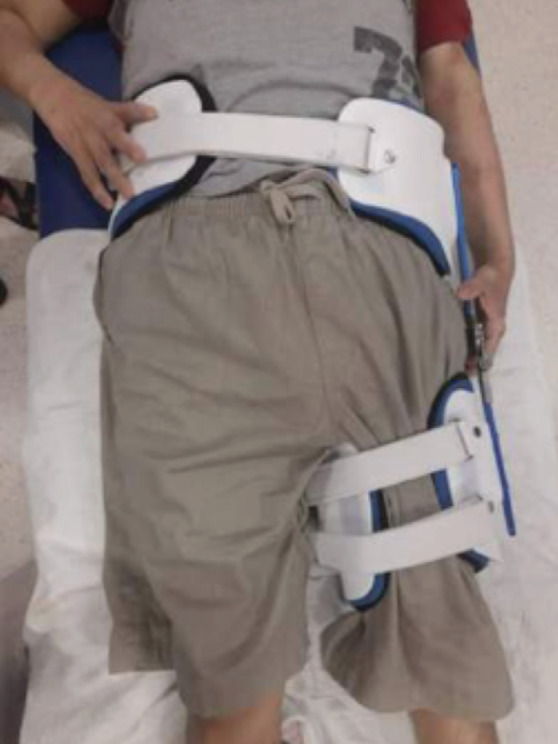
Application of femoral functional brace over left femur.

Patient and family members was counselled for intramedullary nailing and cancellous bone graft augmentation; however, they were not keen for any further surgical interventions and opted for non-operative treatment. Thus, we applied a femoral functional brace (Fig. 2) with the hope that the patient may be able to ambulate at least via walking frame with good rehabilitation services.

Preparation of the femur functional brace was done by our rehabilitation team through co-management with our team. We applied the patient on a Hip Brace Support System functional femoral brace. Femoral brace was made of a plastic rim with pad support consisting of a waist and thigh component. The waist belt size fits most waists 25”–53’’ and it is measured by measuring the waist at the level of the navel. The waist belt component is applied around 2.5cm above widest part of the hip. The thigh component was applied on the thigh up to the desired position (fracture site) with the hinge at the level of hip joint. This brace also allows controlled hip flexion and extension adjustments in 15° increments. The reason behind this design is for immobilisation and stabilisation of fracture site while allowing joint motion to prevent stiffness. It also restrains soft tissue expansion, directing force equally in all directions internally during muscle contraction giving a pseudo hydraulic environment which stabilises the fracture.

**Fig. 3:**
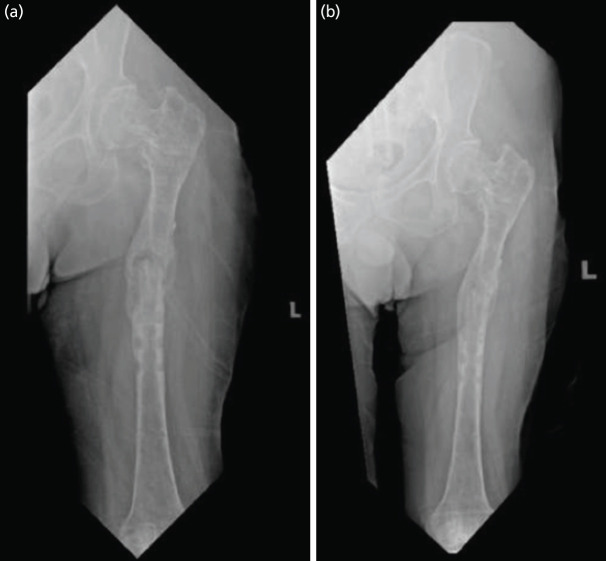
AP radiograph of Left femur showing (a) good union at fracture site after five months, (b) good consolidation at fracture site after eight months after femoral functional brace.

Following the application of the functional femur brace, no specific rehabilitation protocol was applied as no such protocol was available. Therefore, we decided to plan weight bearing based on serial assessment of the fracture union through radiographs investigation. Range of motion of the hip whilst on the brace was allowed as tolerated and was monitored and controlled well by his caregivers. The weight bearing decision was mainly decided by our team as we feared that the functional brace may not be as stable as an external fixator and might lead to over mobilisation of the fracture site. Immediately following discharge, we kept him on a wheelchair. Within a month, he was able to stand and move around using a walking frame, while keeping weight off the affected limb, with the assistance of two people. This further progressed to only needing a single person to assist him. Serial radiographs showed good progress of union and by five months post-op, the fracture site was deemed to be united (Fig. 3a). At that follow-up, we decided to allow him protected weight bearing or partial weight bearing. After eight months of follow-up, radiographs exhibited good consolidation over the fracture site (Fig. 3b), and he was allowed full weight bearing during ambulation. The range of motion of his hip was a flexion of 0°-100°, extension of 0°-10°, abduction of 0°-30°, adduction of 0°-5°, internal rotation of 0°-10° and external rotation of 0°-30°. Prior to discharge he was ambulating with wheelchair, and by one month, he was able to walk (non-weight bearing over left lower limb) with walking frame with two-man support. Gradually he was able to stand from sitting position with arm on walking frame only with one assistant. Serial radiographs in a period of five months showed fracture healing with good union (Fig. 3a) and by then, he was able to full weight bear and ambulate using walking frame unaided while wearing femoral functional brace. By eight months of treatment with functional brace, radiograph showed good consolidation (Fig. 3b) and by nine months the functional brace was completely removed. At this point, the patient was able to ambulate well with walking frame and having a final hip range of motion of flexion of 0°-100°, extension of 0°-10°, abduction of 0°-30°, adduction of 0°-5°, internal rotation of 0°-10° and external rotation of 0°-30°.

## Discussion

Non-union of the femoral shaft fracture represents a challenge for orthopaedic surgeons and a serious socioeconomic problem for the patients mainly due to blood supply damage or inadequate fracture stability^2^. Recently in 2016, a bone healing and non-union theory was advocated. The key to this theory is the concept that the tissue that forms in and around fracture site should be considered as a specific functional entity (bone healing unit). It suggests that majority of non-union heals if a correct mechanical environment is introduced without the need for biological adjuncts (E.g.: bone graft) in the surgery^2^. An optimal condition must be created for treatment of non-union to succeed.

The principle behind functional bracing is that it maintains that rigid immobilisation of fractures of long bones is unphysiological and that movement at the site of a fracture during functional activities encourages osteogenesis^3^. Research findings indicate that the strength of callus production at the site of a fracture, where movement is present, is higher compared to cases involving rigid fixation or immobilisation. The local irritation of the soft tissues surrounding a fracture leads to vascular invasion that ultimately contributes to a higher degree of osteogenesis in which does not occur with rigid fixation. It has also been observed that the osteoblasts which form in non-immobilised fractures show greater activity^1^.

Fortunately, in our case, the fracture union was achieved after a period of five months with the application of a functional brace. Treatment period was comparable to that reported by others using functional brace in treatment of tibia non-union^1^. We postulate that the fracture still managed to achieve union due to a few reasons. The decision of not opening the fracture site again most likely has led to preservation of essential regeneration cells and blood supply to the fracture site. This non-disruptive approach to the fracture site further stimulates formation of good blood supply to the periosteum and endosteum. Functional fracture brace provides fracture alignment stability without rigid immobilisation. Sarmiento in 2000 and 2003, and Balfour in 1982 shows that functional brace acts by compressing the surrounding muscle and soft tissue, creating a tube that provides adequate fracture stability that permits early joint motion above and below fracture site^1,3,4^. Besides preserving biologic function, it also provides “controlled” motion at the fracture site to stimulate osteogenesis. In 2010, Takigami *et al* reported a case of successful treatment of delayed femur union using functional brace. Insertion of a non-vascularised fibula bone graft further increased the stability around the fracture site^5^. Therefore, we believe this relatively good stability coupled with excellent preservation of the biological environment with good rehabilitation and weight bearing control had led to an effective union of the bone. However, further research in determining if these postulations are true would be beneficial. The efficacy of this therapeutic intervention indicates that functional bracing, a method that aims to preserve biological function, should be contemplated as a viable option for managing non-union.

As a conclusion, functional bracing can be considered an option for treating femur non-union as it provides adequate mechanical stability whilst preserving the biological environment to promote fracture healing. It also has advantages of maintaining muscle function and joint motion while reducing costs and avoids surgical complications of internal fixation. Success of this treatment also can be credited to the patient’s compliance to rehabilitation protocol as well as good family support.

## References

[1] Sarmiento A, Burkhalter WE, Latta LL (2003). Functional bracing in the treatment of delayed union and nonunion of the tibia.. Int Orthop..

[2] Elliott DS, Newman KJ, Forward DP, Hahn DM, Ollivere B, Kojima K (2016). A unified theory of bone healing and nonunion: BHN theory.. Bone Joint J..

[3] Sarmiento A, Zagorski JB, Zych GA, Latta LL, Capps CA (2000). Functional bracing for the treatment of fractures of the humeral diaphysis.. J Bone Joint Surg Am..

[4] Balfour GW, Mooney V, Ashby ME (1982). Diaphyseal fractures of the humerus treated with a ready-made fracture brace.. J Bone Joint Surg Am..

[5] Takigami I, Ohara A, Matsumoto K, Fukuta M, Shimizu K (2010). Functional bracing for delayed union of a femur fracture associated with Paget's disease of the bone in an Asian patient: a case report.. J Orthop Surg Res..

